# Human enhancement and personality: A new approach towards investigating their relationship

**DOI:** 10.1016/j.heliyon.2022.e09359

**Published:** 2022-04-30

**Authors:** Sandra Grinschgl, Zadaf Tawakol, Aljoscha C. Neubauer

**Affiliations:** Department of Psychology, University of Graz, Austria

**Keywords:** Human enhancement, Transhumanism, Values, Openness, Dark triad

## Abstract

With the rise of new technologies, also human enhancement is widely discussed. Especially the philosophical movement “transhumanism” urges for creating “better humans” by applying different enhancement methods, namely: pharmacological, current-based, and genetic enhancement as well as mind uploading. While the first three aim at enhancing human characteristics, mind uploading promises immortality by uploading one's brain onto an external storage medium. In the present study, we adapted the method of divergent thinking tasks to assess individuals' assumptions about enhancement methods. These were rated regarding their negativity/positivity and societal-/individual-orientation and then tested whether they are predicted by basic human values and selected personality traits. While individuals' values were not related to their assumptions about enhancement, openness predicted more negative assumptions about most enhancement methods, and a higher intellect predicted more societal-oriented assumptions about genetic enhancement. Furthermore, higher grandiose narcissism predicted more negative assumptions about current-based enhancement and higher psychopathic tendencies predicted more positive assumptions about genetic enhancement. Additionally, higher Machiavellianism predicted more individual-oriented assumptions about pharmacological and current-based enhancement. However, all relationships were of small effect size. We urge for further psychological research in this increasingly relevant field.

## 1 Introduction

In the 21^st^ century, fears but also possibilities for the future of humanity are in the centre of public debates. As new technologies develop further, scientists, politics, and the public are discussing the impact of this progress. A philosophical movement especially dealing with humanity's future is the transhumanism. The core idea of transhumanism is the transformation of humankind by the means of technological progress (Hansell and Grassie, 2011). Different enhancement methods are promoted to create “better humans”. Although Transhumanism itself might not (yet) be a well-known movement within the general population, the topic of human enhancement is widely discussed in media nowadays. For instance, searching the New York Times for the term “human enhancement” yields around 14.500 articles (as of 15^th^ October 2021) and Netflix streams several series/episodes dealing with human enhancement (e.g., Black Mirror, Unnatural Selection). Thus, most individuals within the general population should have heard about human enhancement and formed opinions about it. However, although discussions on human enhancement are omnipresent, empirical psychology is practically absent in these discussions (as compared to other disciplines; see Neubauer, 2021). It is thus mostly unclear how laypersons view human enhancement scenarios. In the present study, we took a first step towards investigating individuals' attitudes (i.e. different assumptions) towards enhancement scenarios from a psychological perspective. More specifically, we present a first-try exploratory study investigating the relationship of individuals' values and selected personality traits with their assumptions about enhancement methods.

Human enhancement scenarios are well known from science fiction movies and novels for decades. However, they are not exclusively futuristic; rather, 21^st^ century scientific discoveries represent tiny steps on a longer path toward a possible transhumanistic world with enhanced individuals (Walker, 2011). The four most frequently proposed enhancement methods are pharmacological enhancement, current-based enhancement, genetic enhancement, and mind uploading.

**Pharmacological enhancement** targets the enhancement of the central nervous system. Psychostimulants such as modafinil, methylphenidate, and amphetamine are known to increase cognitive performance, or to change personality-related aspects (e.g., Repantis et al., 2010).

**Current-based enhancement** relies on current-based technologies such as transcranial electric, magnetic, and deep brain stimulation. These technologies have been tested to enhance episodic memory (e.g., Hamani et al., 2008), working memory (e.g., Luber et al., 2007), and intelligence (e.g., Neubauer et al., 2017).

**Genetic Enhancement** refers to the prenatal selection and modification of genetically determined aspects. The CRISPR-Cas9 system (used to selectively cut and replace DNA sequences) opens new possibilities for biology and medicine (Doudna and Charpentier, 2014).

**Mind uploading** describes the idea of digitally replicating the cellular structure of the human brain and uploading it onto an external storage medium, where the replicated brain is emulated (Laakasuo et al., 2018). The EU funded "Human Brain Project" aims at a detailed understanding and replication of the brain (Markram, 2012).

Importantly, transhumanists argue for applying these methods on healthy individuals to, for instance, enhance their abilities and not to treat diseases with them as it is sometimes already done. Although currently successes of enhancement are rather modest, they are developing rapidly and will probably become increasingly popular in the next years (cf. Neubauer, 2021). When making enhancement methods publicly available, regulations might be needed to prevent misuse or an extreme social stratification (e.g., wealthier people might have more money for using enhancement). Thus, investigating individuals’ attitudes towards different enhancement methods could help creating regulations and guidelines for them – already now during the development of these methods and in future when they might be successfully applied.

Qualitative meta-analyses recapitulated laypersons' opinions towards some enhancement methods (Dijkstra and Schuijff, 2016; Schelle et al., 2014). Laypersons' were mostly concerned about medical safety, coercion (i.e. pressure to use enhancement), and fairness (e.g., equal opportunity to use enhancement; Schelle et al., 2014). Arguments against enhancement mainly related to (negative) consequences for the society, whereas arguments in favour related to (positive) consequences for the individual (Dijkstra and Schuijff, 2016). Overall, attitudes towards enhancement were rather negative. Few studies further investigated factors related to individuals' attitudes towards enhancement (e.g., Laakasuo et al., 2018, 2020; Mayor et al., 2020), however, they focused on a single enhancement method rather than comparing them. For instance, Laakasuo et al. (2018) observed that individuals’ acceptance of mind uploading is predicted by science fiction literacy, but not related to values and personality.

Until now, it is widely unclear how individuals' values and personality impact their attitudes about different enhancement methods. From the meta-analysis by Dijkstra and Schuijff (2016), it can be derived that assumptions about enhancement can be qualitatively classified on the dimensions negativity/positivity (N/P) and societal-/individual-orientation (S/I). Thus, we exploratorily tested which values and personality traits predict the N/P and S/I of individuals' assumptions about the four most common enhancement methods. More specifically, we tested individuals’ assumptions about enhancement using the method of a classical divergent thinking task and their basic human values, big five factor openness, and dark triad traits (narcissism, psychopathy, Machiavellianism).

Schwartz (1992) defines values as beliefs coupled to emotions that motivate goal-directed behaviour. These values can be grouped into collectivistic and individualistic interests (Schwartz, 2003) and are associated with personality (e.g., Parks-Leduc et al., 2015). Further, they are supposed to serve as a criterion to evaluate stimuli individuals come across (Schwartz, 2003). Thus, they might also relate to the nature of individuals’ attitudes about different enhancement methods (e.g., more individualistic interests could be associated to more individual-oriented assumptions).

Moreover, we measured individuals' openness, which reflects the capacity and the tendency for cognitive exploration in terms of thought and perceptual processes (DeYoung, 2014). Matthews et al. (2021) suggest that high openness might be beneficial when interacting with new technologies, but only to a certain degree. If technologies are too hard to comprehend, individuals with high openness might get frustrated. Anyhow, openness (and its' two aspects “openness” and “intellect”) could be related to individuals’ attitudes about enhancement.

Furthermore, dark triad traits are known to be associated with utilitarian and self-interested decision-making in dilemmas (e.g., Deutchman and Sullivan, 2018), and valuing individual interests, particularly self-enhancement (Jonason et al., 2015). They are also linked to positive attitudes towards cognitive enhancement (Mayor et al., 2020), and psychopathy as well as Machiavellianism were associated with a higher approval of mind uploading (Laakasuo et al., 2020). Therefore, we assumed that dark triad traits would be related to individuals’ attitudes about enhancement.

Summarized, we exploratorily investigated individual differences in values and selected personality traits regarding attitudes about different human enhancement methods. We were also interested in the relationship of the N/P- and S/I-dimensions themselves. Due to a lack of previous studies on human enhancement from a psychological perspective, we did not prespecify hypothesis with regard to relationships between personality traits/values and individuals’ assumptions about enhancement. First insights on this behalf, however, cannot only inform future studies on this increasingly relevant topic but also provide first indications on which individuals might be willing to use enhancement.

## 2 Method

The study was conducted online via “Unipark”.

### 2.1 Participants

We collected data of 201 participants (mostly recruited using social media platforms), however, two participants were excluded due to incomplete datasets, yielding a final sample of *N* = 199 (*M*_age_ = 26.91, *SD*_age_ = 11.23; 133 females, 66 males). Most participants were university students (148 participants). Participants with a major in psychology were offered course credits for their participation. The study was conducted in accordance with the ethical principles of the Declaration of Helsinki and approved by the ethics committee of the University of Graz, Austria. Further, all participants provided informed consent.

### 2.2 Measures

Descriptive statistics and reliability indicators of all variables can be found on OSF (https://osf.io/uds2c/). We used the German version of each test and calculated means per participant. All tests were administered in the order as described below.

#### 2.2.1 Attitudes about human enhancement

To assess participants’ attitudes towards different enhancement methods we adapted a just-suppose version of the well-known divergent thinking task, in which the participants have to generate ideas on what could happen for a presented scenario. Divergent thinking tasks are originally used to measure creativity (Torrance, 1974; for a more recent use see e.g., Andolfi et al., 2017). In our version of the task, each scenario referred to one of the four enhancement methods. The participants were asked “*What would be the consequences…”*

- “*…if one could influence his/her personality by taking medication?”* (pharmacological enhancement)
- “*…if one could significantly increase his/her intelligence with the help of electrical brain stimulation?”* (current-based enhancement)
- “*…if one could influence human characteristics by selecting or altering DNA before a child is born?”* (genetic enhancement)
- “*…if one could upload his/her brain and thus his/her talents and personality, thoughts, feelings, and memories onto a storage medium true to the original and thus become immortal?”* (mind uploading).

The participants should imagine that these scenarios were true and think about positive and negative consequences of each scenario. Further, they were told that there could be consequences for individuals on the one hand and for the society on the other. The participants wrote down their assumptions with a maximum of ten responses and a time limit of six minutes. The scenarios were presented in a randomized order.^1^

We asked four independent raters to estimate each response on two dimensions using a 5-point Likert scale; namely, N/P [negative (1), rather negative (2), neutral (3), rather positive (4), positive (5)] and S/I [societal-oriented (1), rather societal-oriented (2), neutral (3), rather individual-oriented (4), individual-oriented (5)]. Intraclass correlations (ICC) were satisfying for N/P in all scenarios (all ICCs ≥ .92, for details see https://osf.io/uds2c/). Regarding S/I, ICCs were satisfying for pharmacological and current-based enhancement (ICCs ≥ .72), but for genetic enhancement (ICC = .31) and mind uploading (ICC = .59), one rater scored participants' responses very differently from the other three and was thus excluded from the analyses. Including only three raters, the ICCs were also satisfying for these two scenarios (genetic enhancement ICC = .61; mind uploading ICC = .78). We calculated the average score across (the remaining) raters per participant for each dimension and scenario. The internal consistencies across the four scenarios for each dimension were rather low (all Cronbach's α ≤ .48; for details see https://osf.io/uds2c/), suggesting that they should be analyzed separately. In addition to the N/P and S/I dimension, we calculated a fluency score (i.e. number of responses) per participant for each scenario (as usual for divergent thinking tasks).

#### 2.2.2 Portrait Values Questionnaire

To measure participants' values, we used the Portrait Values Questionnaire (Schmidt et al., 2007). The participants read portraits of 21 persons and rated how similar they are to them on a 6-point Likert scale [very dissimilar (1) to very similar (6)]. Using centered value scores, the ratings can be averaged to ten individual values, grouped into four higher order values and into individualistic and collectivistic interests. We focused on the latter two categories. The internal consistency of individualistic interests was acceptable (Cronbach's α = .70), whereas it was rather low for collectivistic interests (Cronbach's α = .44).

#### 2.2.3 Big Five Aspects Scale

To keep participants' workload low, we solely assessed that big five factor, for which we expected a relationship with assumptions on enhancement. Thus, we measured openness (ten items each for the aspects “openness” and “intellect”), using the Big Five Aspects Scale (Mussel and Paelecke, 2018) with a 7-point Likert scale [completely disagree (1) to completely agree (7)]. For both aspects, the internal consistency was acceptable to good (all Cronbach's α ≥ .78).

#### 2.2.4 Narcissistic Personality Inventory

Grandiose narcissism was measured with the 15-item forced-choice Narcissistic Personality Inventory (Schütz et al., 2004). The internal consistency for this measure was acceptable (Cronbach's α = .77).

#### 2.2.5 Hypersensitive Narcissism Scale

We administered the 10-item Hypersensitive Narcissism Scale to measure vulnerable narcissism (Jauk et al., 2017; Hendin and Cheek, 1997) with a 5-point Likert scale [strong disagreement (1) to strong agreement (5)]. Also for this scale, the internal consistency was acceptable (Cronbach's α = .71).

#### 2.2.6 Levenson Self Report Psychopathy Scale

To assess psychopathic tendencies, we used the Levenson Self Report Psychopathy Scale (Jauk et al., 2016; Levenson et al., 1995), including 26 items with a 4-point Likert scale [strong disagreement (1) to strong agreement (4)]. The internal consistency was acceptable (Cronbach's α = .77).

#### 2.2.7 Machiavellianism Scale

Finally, the participants answered the 18-item Machiavellianism Scale (Henning and Six, 1977) using a 6-point Likert scale [strong disagreement (1) to strong agreement (6)]. This scale had a good internal consistency (Cronbach's α = .83).

## 3 Results

Due to satisfactory ICCs of enhancement scenarios but low alphas of N/P and S/I aggregates, we decided to analyze them separately. We computed Pearson-correlations between all relevant variables and multiple regression analyses. Further, we performed One-Way repeated measures ANOVAs to compare the different enhancement methods regarding their two dimensions and fluency. Statistical tests were two-tailed and the common assumptions were met unless otherwise noted.

### 3.1 Values and personality traits as predictors of attitudes towards human enhancement

Correlations of participants’ assumptions with values/personality traits can be found in Table 1; most were not significant or of small effect size. We observed a negative relationship between openness and N/P regarding pharmacological enhancement, genetic enhancement, and mind uploading. The higher openness was, the more negative were their assumptions about these scenarios. For mind uploading, higher openness was associated with more societal-oriented assumptions. Higher intellect was accompanied by more societal-oriented assumptions about genetic enhancement and more negative ones about mind uploading. Regarding current-based enhancement, higher grandiose narcissism was accompanied by more negative assumptions. Additionally, higher psychopathy and Machiavellianism were related to more individual-oriented assumptions about current-based enhancement. With exception of this latter scenario, more individual-oriented assumptions were related to more positive assumptions about enhancement. For all intercorrelations see https://osf.io/uds2c/.

**Table 1:** Main correlations.

	Individualistic Interests	Collectivistic Interests	Openness	Intellect	Grandiose Narcissism	Vulnerable Narcissism	Psychopathy	Machiavellianism
Pharmacological E. - N/P	-.05	.02	**-.20∗∗**	-.10	-.10	.04	.11	.13
Current-based E. - N/P	-.03	.09	-.14	-.07	-.**16∗**	.10	-.06	.06
Genetic E. - N/P	.07	-.04	**-.22∗∗**	-.05	-.05	-.01	.10	.02
Mind Uploading - N/P	.06	-.04	**-.17∗**	-.**17∗**	-.05	-.02	.04	.01
Pharmacological E. - S/I	.03	-.05	-.04	-.07	-.07	.03	-.04	.08
Current-based E. - S/I	<.01	-.02	.06	-.05	.07	<.01	**.15∗**	.**22∗∗**
Genetic E. - S/I	-.02	-.04	-.05	-.**18∗**	-.08	-.02	-.03	-.01
Mind Uploading - S/I	-.07	.03	**-.15∗**	-.13	-.12	.06	.05	.09

*Note.* E. refers to enhancement; ∗*p* < .05, ∗∗*p* < .01, ∗∗∗*p* < .001; *N* = 199.

To further analyze the overall prediction, we performed multiple regression analyses. The preconditions for these analyses (i.e. linear relationships between predictor and criterion variables, independent errors, no multicollinearity, homoscedasticity, normally distributed errors; Field, 2018) were given. Each enhancement method was analyzed with either N/P- or S/I-dimension as the criterion. To make the analyses comparable, we included the personality traits that significantly correlated with at least *one* criterion as predictor variables (see Table 1). Thus, all analyses were performed with the predictors openness, intellect, grandiose narcissism, psychopathy, and Machiavellianism (see Table 2).

**Table 2:** Multiple regression analyses with standardized models.

**Pharmacological Enhancement – Criterion: N/P**				
**Predictors**	***R***^***2***^	***F***	***β***	***t***
Openness	.06	2.70∗	-.17	-2.33∗
Intellect			<-.01	-0.01
Grandiose Narcissism			-.14	-1.67
Psychopathy			.09	0.89
Machiavellianism			.07	0.69
**Pharmacological Enhancement – Criterion: S/I**				
Openness	.03	1.32	.01	0.70
Intellect			-.07	-0.80
Grandiose Narcissism			-.05	-0.60
Psychopathy			-.18	-1.80
Machiavellianism			.21	2.15∗
**Current-based Enhancement – Criterion: N/P**				
Openness	.06	2.59∗	-.11	-1.57
Intellect			.02	0.22
Grandiose Narcissism			-.18	-2.15∗
Psychopathy			-.14	-1.41
Machiavellianism			.18	1.81
**Current-based Enhancement – Criterion: S/I**				
Openness	.07	2.83∗	.13	1.77
Intellect			-.10	-1.22
Grandiose Narcissism			.06	0.68
Psychopathy			-.03	-0.31
Machiavellianism			.26	2.64∗∗
**Genetic Enhancement – Criterion: N/P**				
Openness	.08	3.17∗∗	-.25	-3.39∗∗
Intellect			.06	0.76
Grandiose Narcissism			-.10	-1.19
Psychopathy			.21	2.17∗
Machiavellianism			-.15	-1.57
**Genetic Enhancement – Criterion: S/I**				
Openness	.04	1.44	-.01	-0.20
Intellect			-.19	-2.29∗
Grandiose Narcissism			.02	0.19
Psychopathy			-.07	-0.76
Machiavellianism			.03	0.33
**Mind Uploading – Criterion: N/P**				
Openness	.05	2.01	-.15	-1.99∗
Intellect			-.15	-1.77
Grandiose Narcissism			.02	0.28
Psychopathy			.05	0.49
Machiavellianism			-.06	-0.64
**Mind Uploading – Criterion: S/I**				
Openness	.05	1.86	-.11	-1.53
Intellect			-.06	-0.70
Grandiose Narcissism			-.12	-1.36
Psychopathy			<-.01	-0.03
Machiavellianism			.10	0.10

*Note.* ∗*p* < .05, ∗∗*p* < .01, ∗∗∗*p* < .001; *N* = 199.

*Pharmacological Enhancement.* We observed that higher openness predicted more negative assumptions about pharmacological enhancement, but in total only 6% of variance could be explained by this model. Further, higher Machiavellianism predicted more individual oriented assumptions, whereas the overall regression model was not significant.

*Current-based Enhancement.* Higher grandiose narcissism predicted more negative assumptions about current-based enhancement with 6% variance explained. Moreover, higher Machiavellianism predicted more individual-oriented assumptions (7% explained variance).

*Genetic Enhancement.* Higher openness and lower psychopathic tendencies predicted more negative assumptions about genetic enhancement (overall 8% explained variance). Regarding S/I, higher intellect predicted more societal-oriented assumptions, but the overall regression model was not significant.

*Mind uploading.* Regarding mind uploading, higher openness predicted more negative assumptions, but the overall model was not significant. When using S/I as criterion, no significant predictors were observed.

### 3.2 Comparisons of attitudes towards human enhancement

To investigate differences between the enhancement methods regarding N/P and S/I, we performed two Greenhouse-Geisser corrected (adjusting violated sphericity) ANOVAs. Participants' assumptions differed across the scenarios in their N/P, *F*(2.80, 554.05) = 9.17, *p* < .001, *ⴄ*_*p*_^*2*^ = .04. Post-hoc *t*-tests showed, that assumptions about pharmacological and genetic enhancement were more negative than about current-based enhancement and mind uploading, all |*t*(198)|s ≥ 3.07, all *p*s ≤ .002, all *ⴄ*_*p*_^*2*^s ≥ .05; no other differences reached significance. Further, participants' assumptions differed with regard to their S/I, *F*(2.83, 561.08) = 136.49, *p* < .001, *ⴄ*_*p*_^*2*^ = .41. Post-hoc *t*-tests showed that assumptions about current-based enhancement were the least individual-oriented compared to the other enhancement methods, all |*t*(198)|s ≥ 14.64, all *p*s ≤ .001, all *ⴄ*_*p*_^*2*^s ≥ .52. Moreover, assumptions about pharmacological enhancement were less individual-oriented than about mind uploading, *t*(198) = -2.33, *p* = .021, *ⴄ*_*p*_^*2*^ = .03; the remaining comparisons were not significant. Another ANOVA showed a significant effect regarding participants’ fluency in response to the scenarios, *F*(3, 594) = 2.69, *p* = .045, *ⴄ*_*p*_^*2*^ = .01. Fluency was lower regarding mind uploading than genetic enhancement, *t*(198) = 2.66, *p* = .008, *ⴄ*_*p*_^*2*^ = .04; the other comparisons were not significant.

## 4 Discussion

The present research was a first attempt to achieve a better understanding of individuals' attitudes towards human enhancement – a topic that is increasingly discussed by experts (e.g., philosophers, biologists) but also needs consideration from the general public (Dijkstra and Schuijff, 2016). We used a novel approach – an adapted divergent thinking task – to investigate individuals’ assumptions about enhancement regarding their N/P and S/I. Due to a low internal consistency across scenarios, we analysed them separately (corroborated also by the fact that relationships between the dimensions and personality traits were not consistent across scenarios). Overall, only a small amount of variance in the N/P- (all *R*^*2*^s ≤ = .08) and S/I-dimension (all *R*^*2*^s ≤ = .07) could be explained. We discuss our findings in detail now.

First, we observed a relationship between N/P and S/I across most enhancement methods. Similar to findings from Dijkstra and Schuijff (2016), a higher individual-orientation of assumptions about pharmacological and genetic enhancement as well as mind uploading was accompanied by more positive assumptions. Thus, pro-arguments towards enhancement seem to refer to individuals whereas contra-arguments seem directed towards the society.

Second, more negative assumptions about pharmacological and genetic enhancement as well as mind uploading were predicted by higher openness. Possibly, as Matthews et al. (2021) hypothesized, individuals high in openness get easily frustrated when future techniques are hard to comprehend. Further, possible apophenic tendencies (i.e. the tendency to see patterns and relationships when in fact there are none; e.g., Blain et al., 2020) and deficits in latent inhibition related to openness (e.g., Peterson et al., 2002) might lead to dystopian and thus negative associations. In contrast to openness, higher intellect predicted more societal-oriented assumptions about genetic enhancement. Here, consequences for the society might be weightier than in other scenarios (i.e. gene technology opens many new possibilities but is not as unrealistic as mind uploading) and evaluating these consequences might be based on high cognitive ability, which is related to the trait intellect (e.g., DeYoung et al., 2014). While we observed interesting (but small) relationships of openness and intellect with assumptions about human enhancement, it must be noted that we did not assess any other Big 5 factors. Investigating all Big 5 factors as predictors of assumptions about human enhancement is a promising approach for further research (e.g., see Schönthaler et al., 2022 for a first study on this behalf).

Third, for dark triad traits, grandiose narcissism was associated with more negative assumptions about current-based enhancement. As current-based enhancement was framed to *“increase intelligence”*, grandiose narcissists might view easy access to higher intelligence in others as a threat to themselves (e.g., threatening the fragile self-esteem of narcissists; Zeigler-Hill, 2006). Further, higher psychopathic tendencies predicted more positive assumptions about genetic enhancement (however, this effect was only present when controlling for other personality traits and not on a simple correlational basis). Thus, psychopathy might play a role when thinking about DNA manipulations of an unborn child to alter its human characteristics. Also, higher Machiavellianism scores were related to more individual-oriented assumptions about pharmacological and current-based enhancement. This finding matches the assumption that individuals high in Machiavellianism are self-centered and aim at achieving their individual goals (e.g., Rauthmann and Will, 2011). Regarding pharmacological enhancement this effect, however, was only visible in the regression model when controlling for other personality traits and not on a correlational level. For current-based enhancement, we observed correlations with psychopathy and Machiavellianism, but only Machiavellianism predicted more individual-oriented assumptions in the regression model. Similarly, also Mayor et al. (2020) and Laakasuo et al. (2020) found correlations of psychopathy and Machiavellianism with the acceptance of cognitive enhancement and mind uploading, but only Machiavellianism predicted higher acceptance. This could be due to the high correlation between psychopathy and Machiavellianism (cf. the concerns about the distinction of these traits; Miller et al., 2017). Altogether, relationships between some dark traits and assumptions about enhancement methods were inconsistent (i.e. only present at one or two enhancement methods). Nevertheless, dark traits might be relevant for individuals’ attitudes towards human enhancement - especially regarding the current-based enhancement scenario which aimed at increasing intelligence, thus being particularly desired by individuals with dark personality (e.g., Rauthmann, 2012).

Fourth, we did not observe any relationship between individuals' values and their assumptions about enhancement; both, when grouped into individualistic vs. collectivistic^2^ interests and higher order values (openness to change, self-transcendence, self-enhancement, and conservation; see https://osf.io/uds2c/). This is especially surprising regarding the value “self-enhancement” as one would expect that individuals valuing self-enhancement might also view human enhancement positively. The evaluation of enhancement methods might therefore either not be based on values generally or not be considered as an effective self-enhancement strategy, yet. Indeed, Laakasuo et al. (2018) observed similar results for individuals’ acceptance of mind uploading.

Fifth and finally, we tested the differences between the enhancement methods regarding their N/P, S/I, and fluency. Assumptions about pharmacological and genetic enhancement were more negative than about mind uploading and current-based enhancement. This might be due to the explicitness of the enhanced domains in the latter case (cognitive ability for current-based enhancement and immortality for mind uploading). In comparison, for pharmacological and genetic enhancement the domains (personality and human characteristics) seem less explicit, thus potentially creating more scope for uncertainty and more negative assumptions. Additionally, the topics of “smart drugs” and “designer babies” might be more familiar to individuals and therefore negatively associated. Also, the technological component underlying current-based enhancement and mind uploading and the medical component in pharmacological and genetic enhancement might lead to different levels of trust in and evaluation of methods. We further observed that assumptions about current-based enhancement were the least individual-oriented compared to the other methods. Moreover, fluency was lower for mind uploading than genetic enhancement (with the other two scenarios lying in between). As the most unrealistic scenario, assumptions about mind uploading might be particularly difficult to generate, resulting in low fluency.

In sum, our results suggest that some of the investigated personality traits indeed play a role for individuals’ attitudes towards human enhancement, whereas basic human values seem irrelevant for them. However, it must be considered, that the observed effects were of a small effect size, thus we cannot draw strong conclusions regarding the interplay of personality and attitudes towards enhancement methods. This might be due to the novelty of enhancement and thus a lack of strong opinions towards them. This, too, shows in the fact that N/P-scores were usually neutral to negative and S/I-scores were neutral to individual-oriented (see https://osf.io/uds2c/). It should also be noted, that most of our participants were university students - a relatively homogeneous sample. Collecting data of participants with a wider demographic range might result in more diverse opinions about human enhancement. Moreover, one could argue that performing studies with a larger sample size might reveal additional relationships due to an increased statistical power. However, simulation studies show that correlation effects already stabilize when approaching 250 participants (Schönbrodt and Perugini, 2013), which our study satisfies. Additionally, effects that are too small to be detected by the present sample, might not be of a high social and scientific relevance (see Kirk, 2001, for a discussion on useful effect sizes). Anyhow, a replication of the present study as well as preregistered follow-up studies are necessary before strong conclusion on the relationship of personality traits and attitudes towards enhancement can be drawn.

The interpretation of our study is also limited by the fact that we only assessed participants' personality with the Big 5 factor openness and antisocial traits (i.e. the dark triad). In addition, since the relationships we found were weak, they would not survive an alpha correction. Nevertheless, other personality-related factors might influence assumptions about enhancement. Human enhancement can be seen as a many-sided concept with different positive and negative aspects, thus also factors related to enhancement might be versatile and exceed the tested personality traits. For instance, as an anonymous reviewer suggested, the pursuit for sustainability might predict positive assumptions towards enhancement. Furthermore, previous studies suggested that factors related to one's interests, such as science fiction literacy, might have a strong impact on assumptions about enhancement (see Laakasuo et al., 2018). We, thus, strongly urge for further studies testing different factors that might be to related to individuals' assumptions about different human enhancement methods. In addition, the differing methodological approaches we used (divergent thinking tasks vs. self-report measures) might have attenuated the tested relationships. Therefore, it might be advisable to construct a standardized questionnaire for assessing attitudes towards enhancement. Nevertheless, the present study is an important starting point towards more systematic research investigating the relationship between values, personality, and human enhancement scenarios.

Our study shows that assumptions about human enhancement (and their relationships with single personality traits) vary depending on the investigated enhancement scenario. From a practical perspective, enhancement methods that are viewed as more positive might be developed and provided to the public faster than methods that are viewed as more negative. Furthermore, individual differences with regard to assumptions about enhancement could be considered when designing enhancement methods as well as providing guidelines for using them. As enhancement (e.g., the frequent consumption of smart drugs or regular stimulations of one's brain) might induce negative side-effects (Hills and Hertwig, 2011), guidelines might be needed in order to avoid or minimize negative outcomes. Onto this account, knowledge about individuals who are drawn to use specific enhancement methods could help to specifically target them with such guidelines. The rise of new technologies and transhumanistic discussions urge for a systematic investigation of the interaction between this progress and humankind. Empirical psychology needs to catch up to answer the many unanswered questions about our future with technology and enhancement (Neubauer, 2021). Possible links with personality traits might be highly relevant when human enhancement scenarios become real.

## Declarations

### Author contribution statement

Sandra Grinschgl: Analyzed and interpreted the data; Contributed reagents, materials, analysis tools or data; Wrote the paper.

Zadaf Tawakol: Conceived and designed the experiments; Performed the experiments; Contributed reagents, materials, analysis tools or data; Wrote the paper.

Aljoscha C. Neubauer: Conceived and designed the experiments; Contributed reagents, materials, analysis tools or data; Wrote the paper.

### Funding statement

This research did not receive any specific grant from funding agencies in the public, commercial, or not-for-profit sectors.

### Data availability statement

Data will be made available on request.

### Declaration of interests statement

The authors declare no conflict of interest.

### Additional information

No additional information is available for this paper.

## Footnotes

1 In the original version of this task measuring creativity (Torrance, 1974), the participants had a time limit of 5 min and no limit of responses. As our scenarios were more complex than the original ones, we decided to slightly increase the time limit. A pilot study with a think-aloud method (*N* = 8), proved this method to be suitable and helped us phrasing the instruction as well as scenarios so that they are easily understandable.

2 Please note, that for collectivistic interests the internal consistency was rather low, suggesting that the included values do not refer to the same construct. Nevertheless, also when looking at the single values no consistent relationships with assumptions about enhancement were observed (for details see https://osf.io/uds2c/).
